# The Characteristic of Transbronchial Lung Cryobiopsy in the Pathological Diagnosis of Hypersensitivity Pneumonitis

**DOI:** 10.3390/jcm12113663

**Published:** 2023-05-25

**Authors:** Shuuhei Ohno, Yoshiaki Zaizen, Goushi Matama, Tomonori Chikasue, Saeko Tokisawa, Masaki Okamoto, Kazuhiro Tabata, Masaki Tominaga, Jun Akiba, Kiminori Fujimoto, Junya Fukuoka, Tomoaki Hoshino

**Affiliations:** 1 Division of Respirology, Neurology and Rheumatology, Department of Medicine, Kurume University School of Medicine, 67 Asahi-machi, Kurume, Fukuoka 830-0011, Japan; ohno_shuuhei@med.kurume-u.ac.jp (S.O.); matama_goushi@med.kurume-u.ac.jp (G.M.); tokisawa_saeko@med.kurume-u.ac.jp (S.T.); okamoto_masaki@med.kurume-u.ac.jp (M.O.); hoshino@med.kurume-u.ac.jp (T.H.); 2 Department of Pathology Informatics, Nagasaki University Graduate School of Biomedical Sciences, 1-7-1 Sakamoto, Nagasaki 852-8501, Japan; fukuokaj@nagasaki-u.ac.jp; 3 Department of Radiology, Kurume University School of Medicine, 67 Asahi-machi, Kurume, Fukuoka 830-0011, Japan; chikasue_tomonori@med.kurume-u.ac.jp (T.C.); kimichan@med.kurume-u.ac.jp (K.F.); 4 Department of Respirology and Clinical Research Center, National Hospital Organization Kyushu Medical Center, 1-8-1 Jigyouhama, Chuo-ku, Fukuoka 810-8563, Japan; 5 Department of Pathology, Kagoshima University Graduate School of Medical and Dental Sciences, 8-35-1 Sakuragaoka, Kagoshima 890-8544, Japan; tabatak.kufm@gmail.com; 6 Department of Community Medicine, Kurume University School of Medicine, 67 Asahi-machi, Kurume, Fukuoka 830-0011, Japan; tominaga_masaki@med.kurume-u.ac.jp; 7 Department of Pathology, Kurume University School of Medicine, 67 Asahi-machi, Kurume, Fukuoka 830-0011, Japan; akiba@med.kurume-u.ac.jp

**Keywords:** airway-centered interstitial fibrosis, bronchoscope, hypersensitivity pneumonitis, interstitial lung disease, pulmonary fibrosis

## Abstract

Background: Transbronchial lung cryobiopsy (TBLC) has widely used for the diagnosis of diffuse lung disease. However, it remains unclear whether TBLC is useful for the diagnosis in hypersensitivity pneumonitis (HP). Methods: We investigated 18 patients who underwent TBLC and were diagnosed with HP based on pathology or multidisciplinary discussion (MDD). Of the 18 patients, 12 had fibrotic HP (fHP), 2 had non-fibrotic HP (non-fHP) diagnosed with MDD. The remaining 4 patients were diagnosed with fHP by pathology but not by MDD because of clinical features. The radiology and pathology of these cases were compared. Results: All patients with fHP showed radiological findings of inflammation, fibrosis, and airway disease. Conversely, pathology showed fibrosis and inflammation in 11 of 12 cases (92%), but airway disease was significantly less common with 5 cases (42%) (*p* = 0.014). Non-fHP showed inflammatory cell infiltration mainly in the centrilobule on pathology, which was consistent with radiology. Granulomas were found in 5 patients with HP (36%). In the non-HP group, airway-centered interstitial fibrosis was observed in 3 patients (75%) with pathology. Conclusions: The pathology with TBLC is difficult to evaluate airway disease of HP. We need to understand this characteristic of TBLC to make a MDD diagnosis of HP.

## 1. Introduction

Hypersensitivity pneumonitis (HP) is an interstitial pneumonia that occurs through immunological mechanisms in individuals exposed to specific or unspecified antigens [1]. It is caused by multiple causative agents present in workplace or home, including microbial, animal, and plant proteins, as well as organic and inorganic chemicals [2]. Patients with chronic HP with a strong histopathologic tendency toward fibrosis have a significantly shorter survival time than patients without fibrosis [3]. The ATS/JRS/ALAT clinical practice guideline for HP published in 2020 classify HP into a fibrotic or non-fibrotic type [4]. According to this guideline, the diagnostic certainty of HP depends on high-resolution computed tomography (HRCT) findings, exposure history, lymphocyte proliferation by bronchoalveolar lavage (BAL), and pathological findings. HRCT findings in non-fibrotic HP (non-fHP) are characterized by some findings; parenchymal infiltration suggestive of inflammation, such as mosaic pattern, ground-glass opacity, and consolidation; and airway lesions, such as air trapping, abnormal lung parenchyma in lobule-centered nodules <5 mm, and lung cysts. In fibrotic HP (fHP), HRCT findings are characterized by the presence of coexisting fibrosis of the lungs, centrilobular nodules or three-density patterns suggestive of airway disease, and ground-glass opacity or mosaic patterns suggestive of inflammatory findings. The pathology of non-fHP is characterized by lymphocyte-predominant centrilobular inflammation, chronic bronchiolitis, and granulomatous inflammation, which reflect airway involvement and inflammatory findings, whereas that of fHP is characterized by the presence of patchy fibrosis under the pleura suggestive of chronic fibrotic interstitial pneumonia, as well as airway lesions such as airway-centered interstitial fibrosis (ACIF) and granulomas.

Transbronchial lung cryobiopsy (TBLC) is a relatively new method for obtaining lung tissues for the pathological diagnosis in interstitial lung disease [5,6]. This method allows for the collection of tissue samples approximately 10–30 mm^2^ larger than transbronchial lung biopsies and is relatively free of artifacts such as nuclear fragmentation and alveolar collapse. TBLC can diagnose diffuse lung disease in 70–80% of cases and is particularly accurate and efficient for the diagnosis of UIP [7,8]. Multidisciplinary discussion (MDD) has been reported to improve diagnostic accuracy [9]. According to the latest idiopathic pulmonary fibrosis (IPF) diagnostic guideline, surgical lung biopsy (SLB) is the gold standard for the pathological diagnosis of diffuse lung disease, but TBLC with a relatively low complication risk is an alternative to SLB in some centers [6,10].

For TBLC to replace SLB as the gold standard for the pathological diagnosis of diffuse lung disease, it is necessary to understand how pathology findings in TBLC differ from those in SLB. But the ATS/JRS/ALAT clinical practice guideline for HP [4] describe pathology findings based on SLB, but do not consider whether this is equally applicable to TBLC. It is also necessary to verify which diseases show high and which diseases show low concordance rate between TBLC and surgical lung biopsy. However, TBLC and SLB are not easily performed in the same patient, as shown in the COLDICE study [9]. Therefore, we examined the accuracy of pathological diagnosis compared to MDD diagnosis, the gold standard for diagnosis, and found that fHP and CTD-ILD were significantly less sensitive than IPF in pathological diagnosis based on pathological findings alone, without clinical or radiological information [11]. In the current study, we investigated the usefulness of TBLC in diagnosing HP using diagnostic guideline by comparing histopathological findings, radiological findings, and MDD diagnoses [12].

## 2. Materials and Methods

### 2.1. Study Subjects

This study was conducted in accordance with the tenets of the Declaration of Helsinki and approved by the Nagasaki University Hospital Clinical Research Ethics Committee (No. 21081607). Informed consent was obtained from all the patients.

**Figure 1:**
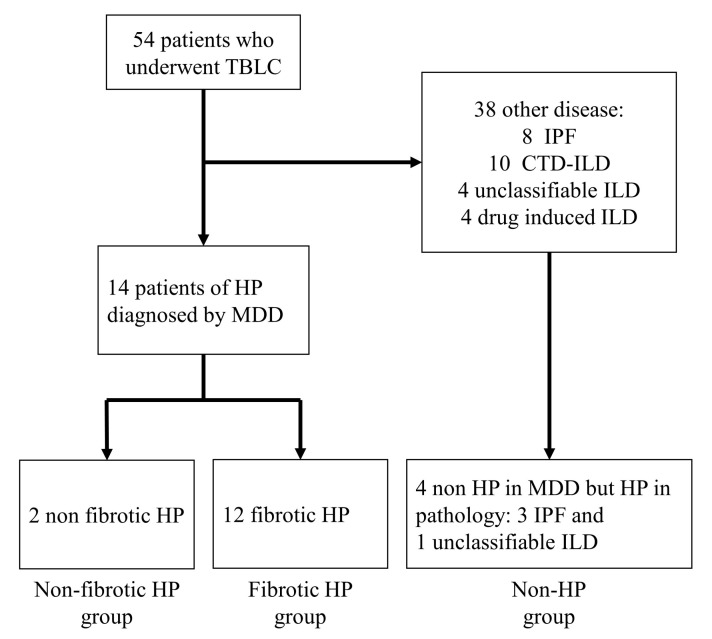
Flowchart of study selection.

This retrospective cross-sectional study investigated 54 patients who underwent TBLC at our clinic between April 2020 and August 2021. Fourteen of the 54 patients were diagnosed with HP based on the official ATS/JRS/ALAT Clinical Practice Guideline [4]. In addition, we investigated 4 cases as non-HP group who diagnosed with fHP in pathology but not in MDD because of clinical feature, with no episodes of antigen inhalation, no lymphocytosis in BAL, and no effectiveness of antigen avoidance (Figure 1). For these 18 patients, patient background at diagnosis, blood test, pulmonary function test, and bronchoalveolar lavage fluid test results were collected from medical records. We also collected information on chest HRCT and histopathological findings as well as diagnoses of MDD made based on these findings.

### 2.2. The Method of TBLC

A flexible BF-1TQ290 bronchoscope (Olympus Corporation, Tokyo, Japan) and a 1.9-mm cryoprobe (Erbe Elektromedizin, Tübingen, Germany) were used for TBLC. All patients underwent intubation with a flexible endotracheal tube and maintained spontaneous respiration under midazolam and fentanyl sedation, the standard protocol in our country. Continuous monitoring of pulse oxygen saturation, blood pressure and electrocardiogram were performed during the entire the examination. The cryoprobe was inserted through the working channel of the flexible bronchoscope, placed under the pleura 1–3 cm away from the pleura under fluoroscopy, and activated for 6–7 s with the 1.9 mm probe.

### 2.3. Evaluation of HRCT Findings Based on HP Diagnostic Guideline

Two radiologists (TC and KF) with expertise in interstitial lung disease (ILD) performed the radiological diagnoses by consensus. The most recent HRCT in which TBLC was performed was evaluated. The guideline described three types of imaging findings: fibrosis, airway disease, and parenchymal infiltration which suggestive of inflammation [4]. Fibrosis includes findings of chronic fibrotic interstitial pneumonia, traction bronchioloectasis, and honeycombing lung; airway disease includes centrilobular nodules and three-density pattern; and parenchymal infiltration includes diffuse ground-glass opacity and consolidation. The HRCT findings of mosaic attenuation suggest parenchymal infiltration, whereas the guideline also described as airway disease in fHP [4]. Therefore, the HRCT findings of mosaic attenuation is considered parenchymal infiltration, and also considered airway disease only in fHP. We assessed these findings and integrated them to determine the presence of fibrosis, airway disease, and parenchymal infiltration/inflammation by radiology. In addition, we evaluated diagnostic confidence based on guideline according to each finding [4].

### 2.4. Pathological Diagnosis

Pathological diagnosis was made by the consensus of three respiratory pathologists (YZ, KT, and JF). Tissues obtained with TBLC were stained using Hematoxylin and eosin (HE), and Elastica van Gieson (EVG). Stained pathology slides were processed into whole-slide images (WSI) using a NanoZoomer S360 (Hamamatsu Photonics K.K., Shizuoka, Japan) at 200×. Three types of pathological findings were evaluated based on the WSI: fibrosis, airway lesions, and inflammation. Fibrosis included findings of chronic fibrotic lesion that suggests a UIP or nonspecific interstitial pneumonia (NSIP) pattern; airway disease included ACIF, peribronchiolar metaplasia (PBM), and bridging fibrosis; and inflammation included lymphocytic infiltration, organizing pneumonia, and granuloma. Poorly formed granuloma were defined in this study as suggestive of both inflammation and airway disease, since they form with inflammation but are also often seen along the airway. On the basis of these findings, we evaluated the presence of fibrosis, airway disease, and inflammation by pathology. Diagnostic confidence was also assessed according to the guideline [4].

### 2.5. Comparison between Radiological and Pathological Diagnosis

We compared the pathological diagnosis with TBLC with the radiological diagnosis for the three categories (fibrosis, airway disease, and inflammation) for each patient. For each category, the positive predictive value of the pathology was calculated and compared with that of the radiological diagnosis. Statistical analyses were performed using the positive predictive values for each category. In addition, the diagnostic confidence level in the MDD diagnosis according to the guideline, which referred to the pathological and imaging diagnosis, was obtained to determine whether the pathological diagnosis contributed to the increase in diagnostic confidence.

### 2.6. Statistical Analysis

All numerical data are presented as median values with 25–75% of the interquartile range. The statistical significance in sensitivity between radiology and pathology was analyzed using McNemar’s test; The present study compared radiology and pathology of the same patients, so we chose the McNemar’s test, which is a representative test method for paired samples. Statistical significance was defined as a *p* value of <0.05, and all statistical analyses were performed using JMP 14.0 (SAS Institute, Cary, NC, USA).

## 3. Results

### 3.1. Patient Characteristics

Eighteen patients are enrolled in this study (Figure 1). The characteristics of the 18 enrolled patients are shown in Table 1. Based on MDD, 12 patients were diagnosed with fHP, 2 were diagnosed with non-fHP, and 4 were diagnosed with non-HP with MDD but suspected HP with pathology. The 4 non-HP patients were not diagnosed with HP because of clinical features. Of these 4 patients, 3 were diagnosed with IPF and 1 with unclassifiable ILD. Four non-HP patients later diagnosed as non-HP with MDD again, including clinical course after diagnosis; One patient was diagnosed with IPF with pleuroparenchymal fibroelastosis because the nodular shadow on HRCT was reduced, but fibroelastosis in the upper lobes and UIP lesions in the lower lobes of the lung were significantly progressed. The remaining three patients still had fibrotic lesions, but after quitting smoking, the mosaic and nodular shadows improved on HRCT. Based on these findings, these three patients were diagnosed with IPF or unclassifiable ILD (mixed UIP and NSIP) with smoking-related interstitial pneumonia changes. The median age of the patients was 73 (range, 36–84) years, and 12 were male. Ten patients, eight fHP patients and two non-fHP patients, were suspected to have been exposed to inhaled antigens. Three patients were positive for anti-tricosporon antibody and anti-dove and anti-parrot antibodies. Two or three TBLC samplings were taken in most cases, and most of these cases were taken from two lobes, one from the lower lobe and the other from the upper or middle lobe. However, in three cases (17%), only one TBLC was sampled from the lower lobe due to adverse events of hemorrhage or pneumothorax. In addition, one other patient in the non-HP group had an adverse event of acute exacerbation after the examination. The average diameter of sampling was 6.2 mm.

### 3.2. Radiological Findings and Diagnosis

The radiological findings of the 18 enrolled patients are shown in Table 2. In all cases of fHP, the presence of one or more items was considered positive for each category of fibrosis, airway disease, and inflammation. Honeycombing, suggestive of severe fibrosis, was observed in 4 of the 12 patients with fHP. Three-density pattern, which suggests airway disease and is considered highly specific for fHP [4], was observed in 5 of the 12 patients. In non-fHP patients, there were no findings suggestive of fibrosis. Airway disease and inflammation were observed in all cases, and three-density pattern was observed in 1 of the 2 cases. In the non-HP group, fibrosis and airway disease were observed in all patients. Inflammatory findings were observed in 3 of 4 patients.

The HRCT pattern in the official ATS/JRS/ALAT Clinical Practice Guideline for HP [4] showed that in the fHP group, 6 patients (50%) were determined as typical fHP, and each of the three patients (25%) were determined as compatible with HP and indeterminate for HP. In contrast, 2 patients with non-fHP were diagnosed with typical HP. In the non-HP group, 3 patients (75%) were determined as compatible with HP.

### 3.3. Pathological Findings and Diagnosis

The pathological findings of the 18 enrolled patients are summarized in Table 3. Eleven of the 12 patients (92%) with fHP had findings suggestive of fibrosis. Airway disease was observed in 5 of the 12 patients (42%). PBM, ACIF, and bridging fibrosis, which are not specific to fHP but are often present in fHP, were seen in 5, 3, and 1 of the 12 fHP patients, respectively. Two non-fHP patients showed no fibrotic lesions, but had airway disease and inflammatory findings. The non-HP group showed fibrosis, airway disease, and inflammatory findings in all cases. PBM was present in all patients, and ACIF was present in 3 of the 4 non-HP patients. We determined these findings were due to smoking or micro aspiration.

The pathology pattern in the guideline [4] showed that in patients with fHP, 8 patients (66%) were diagnosed as indeterminate for HP due to a lack of airway disease findings. However, 3 of the 4 patients (75%) in the non-HP group was diagnosed with probable HP due to the presence of airway disease findings.

### 3.4. Comparison between Radiological and Pathological Findings

A comparison of the radiological and pathological findings in the 18 patients is shown in Table 4. Radiologically, fibrosis, inflammation, and airway disease were observed in all patients with fHP. Pathological examination revealed fibrosis and inflammation in almost all cases (11 of 12 cases, 92%). However, airway disease was observed in only 5 of the 12 cases (42%), which was significantly lower than inflammation and fibrosis (*p* = 0.014). Both inflammation and fibrosis were evaluable in the 2 non-fHP patients, but fibrosis was not found by either radiology or pathology.

In the current study, we investigated the diagnostic confidence level in the guideline [4] when only clinical and imaging information, including BAL, was available without referring to pathology and when pathological information was added to the clinical and radiological information. In fHP, the diagnostic confidence without referring to the pathology data was high in 1 (8%), moderate in 4 (33%), low in 5 (42%), and not excluded in 2 cases (17%). Diagnostic confidence with reference to pathology was definite in 3 (25%), high in 3 (25%), moderate in 2 (17%), low in 3 (25%), and not excluded in 1 case (8%). Referring to pathology increased diagnostic confidence in only 5 cases (42%), and in four cases (33%), diagnostic confidence was still below low confidence even after referring to pathology. On the other hand, the 2 cases of non-fHP showed high diagnostic confidence without reference to the pathological data and definite with reference to the pathological data. In the non-HP group, diagnostic confidence without reference to the pathological data was low in one case (25%) and not excluded in 3 cases (75%). However, referring to the pathological data increased the diagnostic confidence to moderate in 3 of the 4 cases (75%).

**Figure 2:**
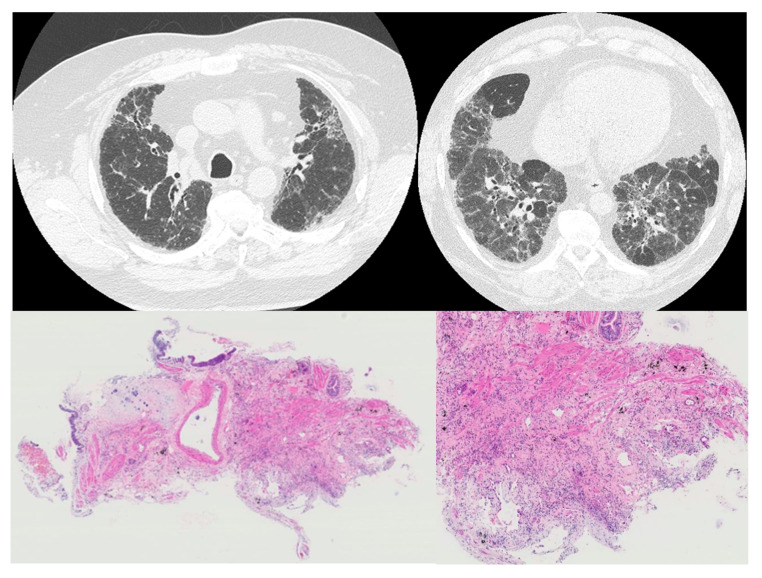
An fHP case in which airway disease was observed radiologically but not pathologically. High-resolution computed tomography (HRCT, (**upper**)) showed centrilobular nodules in the upper lobe and air trappings in the lower lobe. In transbronchial lung cryobiopsy (TBLC) pathology (**lower**), dense fibrosis with architectural destruction was observed in the peripheral of lobules; however, airway disease was not observed in TBLC.

An fHP case in which airway disease was observed radiologically but not pathologically is shown in Figure 2.

## 4. Discussion

In the present study, we evaluated whether pathological findings described in the HP guideline [4] with TBLC were adequate compared with HRCT findings. TBLC was able to diagnose HP with relatively high accuracy for fibrotic lesions and inflammatory findings, whereas airway lesions were significantly underestimated. In the ATS/JRS/ALAT clinical practice guideline for HP [4], the presence of airway disease is a necessary finding in determining the pathology as probable or typical HP. In the guideline-based MDD diagnosis of HP, a pathological diagnosis of probable or typical HP is often necessary to make a diagnosis of HP with high confidence or definitive diagnosis. However, the results of the present study suggest that even if the airway disease are strongly suspected to be present radiologically, the pathological diagnosis with TBLC has a characteristic that makes it difficult to evaluate airway disease. We should be aware of this characteristic when we perform MDD diagnosis.

Tomassetti et al. reported that cryobiopsy was useful in the diagnosis of IPF because the IPF diagnosis was confirmed in all 18 cases wherein IPF was suspected clinically and radiologically even when MDD was performed in addition to pathological diagnosis by TBLC [13]. In this study, the pathological diagnosis of UIP/IPF based on IPF diagnostic guideline showed that although it is difficult to determine a UIP pattern on TBLC, the diagnosis of a probable UIP pattern is sufficient, resulting in a high diagnostic concordance rate of 81.5% (κ = 0.61) for IPF between TBLC and SLB. This suggests that TBLC is an alternative examination method to SLB for IPF diagnosis. In fact, recently updated IPF diagnostic guideline state that TBLC can be a substitute for SLB in some highly specialized institutions [10]. However, it is notable that the COLDICE study [9,14] found 20 cases in which TBLC and SLB did not agree on the MDD diagnosis, with fHP and UIP/IPF being the most common discordant pairs. The MDD diagnosis with TBLC was UIP/IPF, but the MDD diagnosis with SLB was HP in 7 of the 20 cases. One of the reasons for this discordant is that HP could be misdiagnosed as an ILD other than HP, including UIP/IPF, if airway disease was observable in the tissue obtained by SLB but not in that obtained by TBLC, as shown in our study. For this reason, we reported in another study that the sensitivity of pathological diagnosis performed in fHP without adequate clinical and radiological information was 50.8%, which was significantly lower than that of IPF [11]. Furthermore, in a recent CAN-ICE study conducted by Fortin et al. comparing TBLC and SLB in 20 patients prospectively, the sensitivity of HP by TBLC was shown to be low at 51.6% [15]. This CAN-ICE study reported that it is difficult to distinguish between fHP and IPF by TBLC. Churg et al. also reported difficulties in distinguishing between fHP and IPF in pathology specimens of the size taken by TBLC [16]. The current study indicates that one of the reasons why fHP may be misdiagnosed as IPF in pathology by TBLC is the inability to evaluate airway disease. There are, however, still few reports on the pathological diagnosis and MDD diagnosis of hypersensitivity pneumonitis in TBLC. Further additional research is required.

The current study demonstrated that it is difficult to evaluate airway disease in fHP with TBLC. On the other hand, we successfully performed MDD to diagnose fHP by comparison of pathological findings with HRCT findings. In our previous study, the diagnostic accuracy of fHP improved from 50.8% to 81.1% in pathology diagnosis when appropriate clinical and radiological informations were referenced [11]. Zhan et al. also reported that MDD was performed on 46 patients with suspected HP by HRCT and underwent further TBLC, 16 patients could be diagnosed with HP [17]. In this study, ACIF was observed in only two cases (12.5%). If ACIF cannot be observed, we cannot increase our diagnostic confidence from the criteria of the ATS/JRS/ALAT clinical practice guideline for HP [4]. If ACIF cannot be observed, we cannot increase our diagnostic confidence from the criteria of the international diagnostic guidelines. However, if MDD is performed with the recognition that ACIF is difficult to observe at TBLC, MDD with the pathology with TBLC is sufficient to make the MDD diagnosis of HP.

The present study showed that airway disease in fHP may be difficult to evaluate using TBLC; however, bronchiolitis, an airway disease in non-fHP, could be successfully evaluated, although with only 2 validated cases. Because TBLC collects specimens of the transairway, bronchi and bronchioles are frequently included in these specimens. Bronchiolitis which is necessary for the diagnosis of non-fHP may be relatively easy to evaluate with TBLC.

Our study had two limitations. First, this was a single-center retrospective study with a small number of patients. Another limitation was that we compared the diagnostic results of TBLC with those of MDD but not with those of SLB. This is because this was a retrospective analysis in which only TBLC was performed for diagnostic purposes and no additional SLB was performed in clinical practice.

## 5. Conclusions

Histopathologic diagnosis with TBLC is difficult to assess for airway disease in fHP. Consequently, fHP may be misidentified as IPF. Certainly, TBLC may not be well suited for histopathological diagnosis according to HP guidelines. However, when we understand the characteristic of TBLC, which are difficult to assess for ACIF, TBLC can be useful in the diagnosis of HP and adds important information to the MDD.

## Figures and Tables

**Table 1:** Patient characteristics.

	Fibrotic HP	Non-Fibrotic HP	Non-HP
No. of patients	12	2	4
Sex (Female/Male)	5 (43%)/7 (57%)	0/2 (100%)	1 (25%)/3 (75%)
Age (years)	76 (52–84)	44 (36–52)	71 (68–76)
Smoking status (former/current/never)	6 (50%)/0/6 (50%)	1 (50%)/1 (50%)/0	3 (75%)/0/1 (25%)
Reported exposure	8 (67%)	2 (100%)	0
Positive for anti-trichosporon antibody	1 (7%)	0	0
Positive for anti-bird antibody	2 (13%)	0	0
Pulmonary function tests			
FEV1%	81.7 (74.6–89.0)	90.1 (89.5–90.8)	81.1 (78.6–94.9)
%FEV1	76.4 (64.3–96.8)	77.6 (75.9–79.3)	82.6 (81.5–109.7)
FVC(L)	1.98 (1.66–2.45)	3.11 (2.99–3.23)	2.88 (1.70–3.69)
%FVC	73.3 (58.2–97.2)	73.0 (71.5–74.4)	84.3 (68.1–109.3)
%D_LCO_	60.4 (52.3–93.1)	59.4 (54.9–63.9)	78.8 (61.4–93)
BALF			
Total cell count (×10^5^/uL)	1.875 (0.125–6.625)	11.81 (11.12–12.5)	1.25 (0.25–2.375)
Cell segmentation			
Macrophage (%)	81.8 (30–96.8)	13.6 (10.6–16.6)	73.3 (5.6–89.2)
Lymphocyte (%)	16.4 (1.6–67.6)	75.5 (74–77)	12.7 (3.2–43.6)
Neutrophil (%)	3.0 (1–6.8)	8.0 (7–9)	12 (0.6–23.8)
CD4/8 ratio	2.375 (1.15–94)	0.495 (0.3–0.69)	4.175 (2.3–6.05)
Sampling			
Total number (1/2/3)	2/10/0	1/0/1	0/3/1
From upper/middle lobe	9 (75%)	1 (50%)	4 (100%)
From lower lobe	12 (100%)	2 (100%)	3 (75%)
average diameter (mm)	6.0	5.5	7.1
Adverse events			
Hemorrheage	2 (17%)	0	0
Pneumothorax	0	1 (50%)	0
Acute exacerbation	0	0	1 (25%)

BALF, bronchoalveolar lavage fluid; DLCO, diffusing capacity of the lung for carbon monoxide; FEV1%, forced expiratory volume in 1 s; FVC, forced vital capacity; %FEV1, forced expiratory volume % in 1 s; HP, hypersensitivity pneumonitis. Consecutive variables are represented by median value (range).

**Table 2:** Comparison of HRCT findings for each MDD diagnosis.

HRCT Findings	Fibrotic HP (*n* = 12)	Non Fibrotic HP (*n* = 2)	Non-HP (*n* = 4)
Fibrosis	12 (100%)	0 (0%)	4 (100%)
CFIP	12	0	4
Traction bronchiectasis	12	0	4
Honeycombing	4	0	1
Airway disease	12 (100%)	2 (100%)	4 (100%)
Centrilobular nodule	12	2	3
Three-density pattern	5	1	1
Air trapping	10	0	4
Lung cyst	10	0	4
Mosaic attenuation (in fibrotic HP)	12		
Parenchymal infiltration (s/o Inflammation)	12 (100%)	2 (100%)	3 (75%)
Diffuse GGO	7	2	0
Mosaic attenuation	12	1	3
Consolidation	4	0	1
HRCT pattern in GL [4]			
Typical HP	6	2	0
Compatible with HP	3	0	3
Indeterminate for HP	3	N/A	1

CFIP, chronic fibrosing interstitial pneumonia; GGO, ground-glass opacity; GL, guideline; HP, hypersensitivity pneumonitis; HRCT, high-resolution computed tomography; MDD, multidisciplinary discussion; s/o, suggestive of.

**Table 3:** Comparison of histopathological findings for each MDD diagnosis.

Histopathological Findings	Fibrotic HP (*n* = 12)	Non-Fibrotic HP (*n* = 2)	Non-HP (*n* = 4)
Fibrosis	11 (92%)	0 (0%)	4 (100%)
Chronic fibrosing IP	11	0	4
Airway disease	5 (42%)	2 (100%)	4 (100%)
Airway-centered distribution	4	2	3
Airway-centered fibrosis	3	0	3
Peribronchiolar metaplasia	5	0	4
Bronchiolitis	4	2	1
Bridging fibrosis	1	0	0
Poorly formed granuloma	4	1	0
Inflammation	11 (92%)	2 (100%)	4 (100%)
Cellular IP	10	2	4
Organizing pneumonia	4	1	2
Cellular bronchiolitis	2	1	1
Foamy macrophages	3	2	0
Giant cells with cholesterol clefts	2	0	0
Poorly formed granuloma	4	1	0
Pathology pattern in GL [4]			
HP	2	1	0
Probable HP	2	0	3
Indeterminate for HP	8	1	1

GL, guideline; HP, hypersensitivity pneumonitis; IP, interstitial pneumonia.

**Table 4:** Comparison of HRCT and Histopathological findings of fibrotic and non-fibrotic HP.

Dx	Fibrotic Change		Inflammation		Airway Disease		*p* Value	
	Radiology	Pathology	Radiology	Pathology	Radiology	Pathology		
fHP	12 (100%)	11 (92%)	12 (100%)	11 (92%)	12 (100%)	5 (42%)	0.014 *	0.014 **
	92% ^†^		92% ^†^		42% ^†^			
Non-fHP	0	0	2 (100%)	2 (100%)	2 (100%)	2 (100%)	N/A *	1.000 **
	0% ^†^		100% ^†^		100% ^†^			

* Fibrotic changes vs. airway disease. ** Inflammation vs. Airway disease. ^†^ Radiology/pathological ratio. fHP: fibrotic hypersensitivity pneumonia; A *p*-value < 0.05 represented statistical significance by McNemar’s test.

## Data Availability

The datasets used and/or analysed during the current study available from the corresponding author on reasonable request.
